# The Cold Bath as a Means of Diagnosis

**Published:** 1900-12-01

**Authors:** 


### THE COLD BATH AS A MEANS OF DIAGNOSIS.

At a recent meeting held in New York, Dr. Baruch pointed out that the cold bath might be used as a means of diagnosis as well as of treatment in typhoid fever. He said that any patient with a temperature of 103° F. for three days should be first sponged over the trunk and thighs, commencing with water at a temperature of 80° F., gradually reduced to 65° F. He should then be taken to the bathroom and given a bath in water at 90° F., lasting fifteen minutes. Four hours later the bath is to be repeated at a temperature of 85° F.; four hours afterwards at a temperature of 80° F.; and four hours after that at a temperature of 75° F. If in half an hour after the bath the temperature of the body is reduced two degrees, it can be concluded that the case is not one of typhoid fever; but if in half an hour the reduction does not exceed one degree, the diagnosis of typhoid can be made. It will be noted that Dr. Baruch speaks of his patient going to the bathroom, and we believe that in some of the American institutions where the bath treatment is largely practised the custom is for the patients to walk to their baths.

